# Perinatal and newborn care in a two years retrospective study in a first level peripheral hospital in Sicily (Italy)

**DOI:** 10.1186/s13052-019-0751-6

**Published:** 2019-11-29

**Authors:** Gregorio Serra, Vincenzo Miceli, Salvatore Albano, Giovanni Corsello

**Affiliations:** 1 Department of Health Promotion, Mother and Child Care, Internal Medicine and Medical Specialties “G. D’Alessandro”, University of Palermo, Palermo, Italy; 2 “G. Giglio” Hospital Institute Foundation, Cefalù, Italy

**Keywords:** Perinatal mortality, Quality of care, Newborn

## Abstract

**Background:**

Two hundred seventy-five thousand maternal deaths, 2.7 million neonatal deaths, and 2.6 million stillbirths have been estimated in 2015 worldwide, almost all in low-income countries (LICs). Moreover, more than 20 million severe disabilities result from the complications of pregnancy, childbirth or its management each year. A significant decrease of mortality/morbidity rates could be achieved by providing effective perinatal and newborn care also in high-income countries (HICs), especially in peripheral hospitals and/or rural areas, where the number of childbirths per year is often under the minimal threshold recognized by the reference legislation. We report on a 2 years retrospective cohort study, conducted in a first level peripheral hospital in Cefalù, a small city in Sicily (Italy), to evaluate care provided and mortality/morbidity rates. The proposed goal is to improve the quality of care, and the services that peripheral centers can offer.

**Methods:**

We collected data from maternity and neonatal records, over a 2-year period from January 2017 to December 2018. The informations analyzed were related to demographic features (age, ethnicity/origin area, residence, educational level, marital status), diagnosis at admission (attendance of birth training courses, parity, type of pregnancy, gestational age, fetal presentation), mode of delivery, obstetric complications, the weight of the newborns, their feeding and eventual transfer to II level hospitals, also through the Neonatal Emergency Transport Service, if the established criteria were present.

**Results:**

Eight hundred sixteen women were included (age 18–48 years). 179 (22%) attended birth training courses. 763 (93%) were Italian, 53 foreign (7%). 175 (21%) came from outside the province of Palermo. Eight hundred ten were single pregnancies, 6 bigeminal; 783 were at term (96%), 33 preterm (4%, GA 30–41 WG); 434 vaginal deliveries (53%), 382 caesarean sections (47%). One maternal death and 28 (3%) obstetric complications occurred during the study period. The total number of children born to these women was 822, 3 of which stillbirths (3.6‰). 787 (96%) were born at term (>37WG), 35 preterm (4%), 31 of which late preterm. Twenty-one newborns (2.5%) were transferred to II level hospitals. Among them, 3 for moderate/severe prematurity, 18 for mild prematurity/other pathology. The outcome was favorable for all women (except 1 hysterectomy) and the newborns transferred, and no neonatal deaths occurred in the biennium under investigation. Of the remaining 798 newborns, 440 were breastfed at discharge (55%), 337 had a mixed feeding (breastfed/formula fed, 42%) and 21 were formula fed (3%).

**Conclusions:**

Although the minimal standard of adequate perinatal care in Italy is >500 childbirths/year, the aims of the Italian legislation concern the rationalization of birth centers as well as the structural, technological and organizational improvement of health facilities. Therefore, specific contexts and critical areas need to be identified and managed. Adequate resources and intervention strategies should be addressed not only to perinatal emergencies, but also to the management of mild prematurity/pathology, especially in vulnerable populations for social or orographic reasons. The increasing availability and spread of health care offers, even in HICs, cannot be separated from the goal of quality of care, which is an ethic and public health imperative.

## Background

Two hundred seventy-five thousand maternal deaths, 2.7 million neonatal deaths, and 2.6 million stillbirths have been estimated in 2015 worldwide, almost all in low-income countries (LICs) [1]. Moreover, more than 20 million severe disabilities result from the complications of pregnancy, childbirth or its management each year [2]. Mother and child care indicators are internationally recognized as the best to evaluate the quality of health care of a country. Although Italian infant and neonatal mortality rates are among the lowest in the world and constantly decreasing, significant disparities remain disadvantaging insular and southern regions [3]. Therefore, major improvements could be achieved by providing effective perinatal and newborn care also in high-income countries (HICs), especially in peripheral hospitals and/or rural areas [4], where the number of childbirths per year is often under the minimal threshold recognized by the reference legislation. Here we report on a 2 years retrospective cohort study, conducted in a first level peripheral hospital in Cefalù, a small city in Sicily (Italy), to evaluate care provided and mortality/morbidity rates. The goal this study proposes is to improve the quality of care, and the services that peripheral centers can offer.

## Methods

### Study setting

**Fig. 1:**
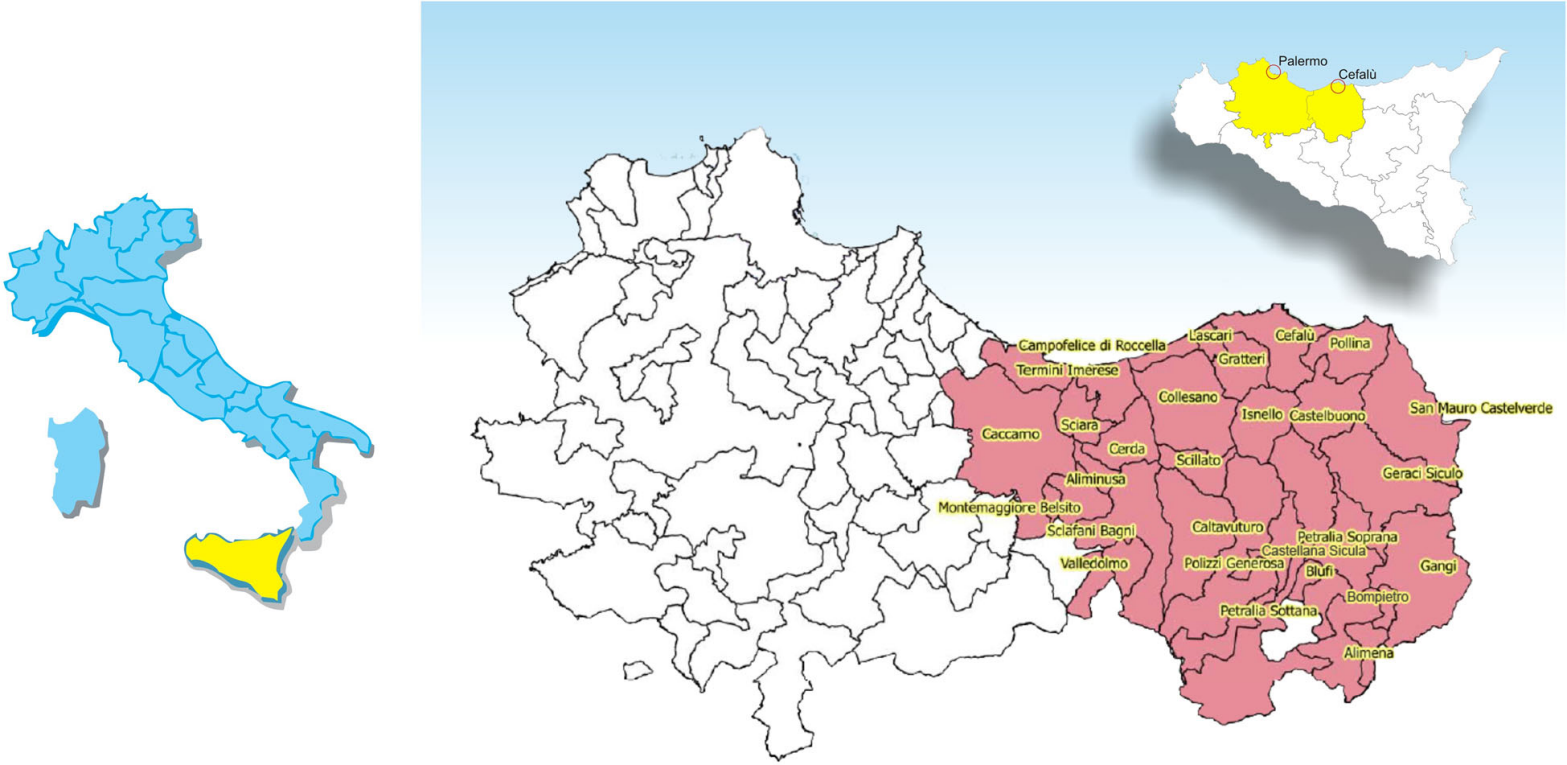
District of “Madonie” (northern Sicily), which is guaranteed by the birth center of the Hospital “G. Giglio” of Cefalù. The predominantly mountainous area spans a surface of around 400 km^2^, 26 municipalities and around 92,000 inhabitants

Cefalù is a town of 14,307 inhabitants [5], nearby Palermo, which is the main city of Sicily. It is located in the northern coast, about 70 km from Palermo (where the Neonatal Emergency Transport Service has its base station), from which it is connected by the highway, with an average travel time of about an hour. The town is included in the “Madonie” area, a short mountain ridge. Such district spans a surface of around 400 km^2^, a population estimated in 2018 around 92,000 inhabitants [6], and 26 municipalities (Fig. 1).

Furthermore, every year, in the summer period, a significant number of tourists triples the population. Within the mountain area the road system is less efficient than the connection with Palermo, with long average travel times, especially in the winter.

**Fig. 2:**
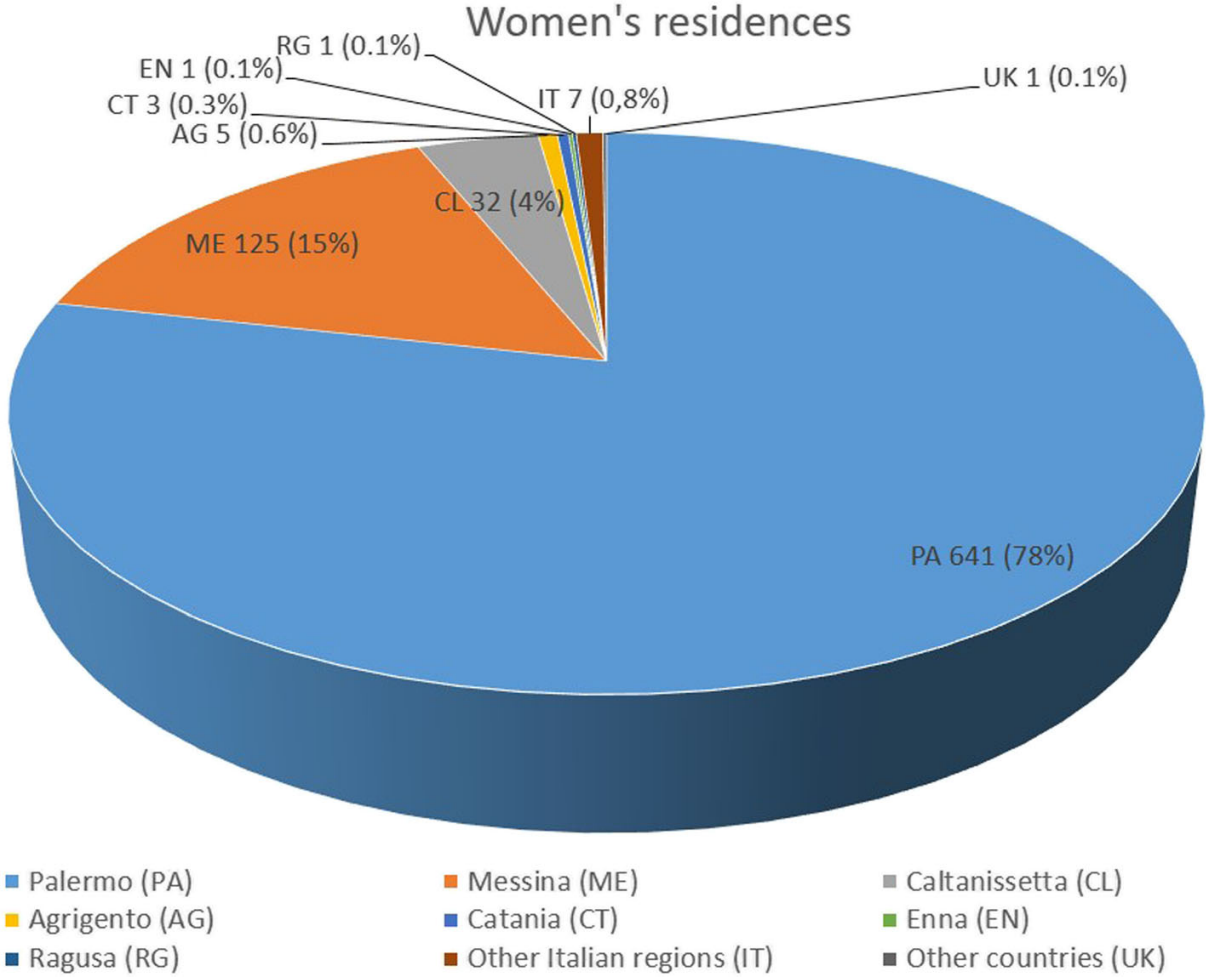
Women’s residences were: 641 province of Palermo (78%), 60 of which outside the “Madonie” district, 125 Messina (15%), 32 Caltanissetta (4%), 5 Agrigento (0.6%), 3 Catania (0.3%), 1 Enna (0.1%), 1 Ragusa (0.1%), 7 other Italian regions (0.8%) and 1 UK (0.1%)

In the study period, a significant number of patients (175, 21% of the total) coming from outside the province of Palermo, especially from the neighboring provinces of Messina and Caltanissetta, referred to our obstetric and neonatal services (Fig. 2), matching those who referred to other birthing hospitals than Cefalù (Palermo, Termini Imerese, etc.). Our staff, during the years under investigation, was composed by 9 midwives, 9 obstetrics and 5 neonatologists/pediatricians, who guaranteed the assistance 24 h/7 days.

### Study population

#### Selection of subjects

We conducted a retrospective cohort study at the Maternity Unit of the Hospital “G. Giglio” of Cefalù, Sicily (Italy). The study took place over a 2-year period, from January 2017 to December 2018. The women included in the study delivered in our unit in such biennium. Among them, 179 (22%) participated to birth training courses, organized during the study period for free, by all the members of the staff and clinical psychologists. The newborns included were those born from these mothers. Except for those transferred to II level centers, they were all (798) discharged between 48 and 72 h of life, and almost all of them (98%, 780 excluding 18 coming from the most distant provinces, and other regions/countries) were re-evaluated at least once within 1 week after discharge. Written informed consent was obtained from each woman at admission, for newborns from both parents after delivery. The study was approved by the Mother and Child Department of the University of Palermo (Palermo, Italy). All procedures performed in this study were in accordance with the ethical standards of the institutional and national research committee, and with the 1964 Helsinki declaration and its later amendments or comparable ethical standards.

#### Data collection

From maternity/neonatal records, the informations analyzed concerned demographic features of the women (age, ethnicity/origin area, residence, educational level, marital status), diagnosis at admission (parity, type of pregnancy, gestational age, fetal presentation), mode of delivery, obstetric complications, the weight of the newborns, their feeding and eventual transfer to II level hospitals, also through the Neonatal Emergency Transport Service, if the established criteria were present [7].

## Results

### Characteristics of subjects

Eight hundred sixteen women were included (age 18–48 years). Their mean age was 33 years, the youngest was 18 years and 11 months old, while the oldest 48 years and 2 months old. 763 (93%) were Italian, 53 foreign (7%, 32 Eastern Europe, 2 Western Europe, 8 Maghreb, 3 USA, 6 Latin America, 2 China). Their residences were: 641 province of Palermo (78%), 60 of which outside the “Madonie” district, 125 Messina (15%), 32 Caltanissetta (4%), 5 Agrigento (0.6%), 3 Catania (0.3%), 1 Enna (0.1%), 1 Ragusa (0.1%), 7 other Italian regions (0.8%) and 1 UK (0.1%) (Fig. 2).

The level of education was thus divided: 209 graduated (26%), 23 short degree (3%), 362 high school (44%), 210 middle school (26%), 12 primary school (1%). Six hundred forty-nine were married (80%), 158 unmarried (19%), 9 separated/divorced (1%). Eight hundred ten were single pregnancies, 6 bigeminal; 802 (98.3%) were naturally obtained, 14 (1.7%) were medically assisted reproductions (7 ICSI and 7 FIVET). Seven hundred eighty-three were at term (96%), 33 preterm (4%, GA 30–41 WG); 434 vaginal deliveries (53%), 382 caesarean sections (47%). One maternal death (1.2‰), related to *postpartum* hemorrhage, and 28 (3%) obstetric complications occurred during the study period. Among these, the most commonly observed were placental abruption and hypertensive crisis. The outcome was favorable for 814 of the 815 remaining women, because of 1 (1.2‰) hysterectomy for placental abruption.

**Fig. 3:**
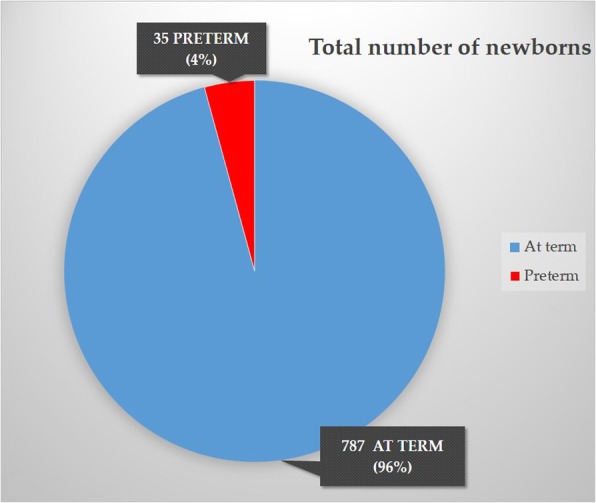
The total number of newborns was 822. 787 (96%) at term (≥37 W), 35 preterm (4%), 31 of which late preterm

The total number of children born to these women was 822, 3 of which stillbirths (3.6‰, 2 intrauterine fetal deaths at 30 and 34 WG and 1 perinatal death at 38 WG). 787 (96%) were born at term (>37WG), 35 preterm (4%), 31 of which late preterm (Fig. 3); 37 (4%) had a birth weight ≤2500 g, 35 (4%) ≥4000 g. Seven hundred seventy-four had cephalic presentation (95.6%), 18 breech (2.2%), 12 bregma (1.5%), 4 shoulder (0.5%) and 2 face (0.2%).

**Fig. 4:**
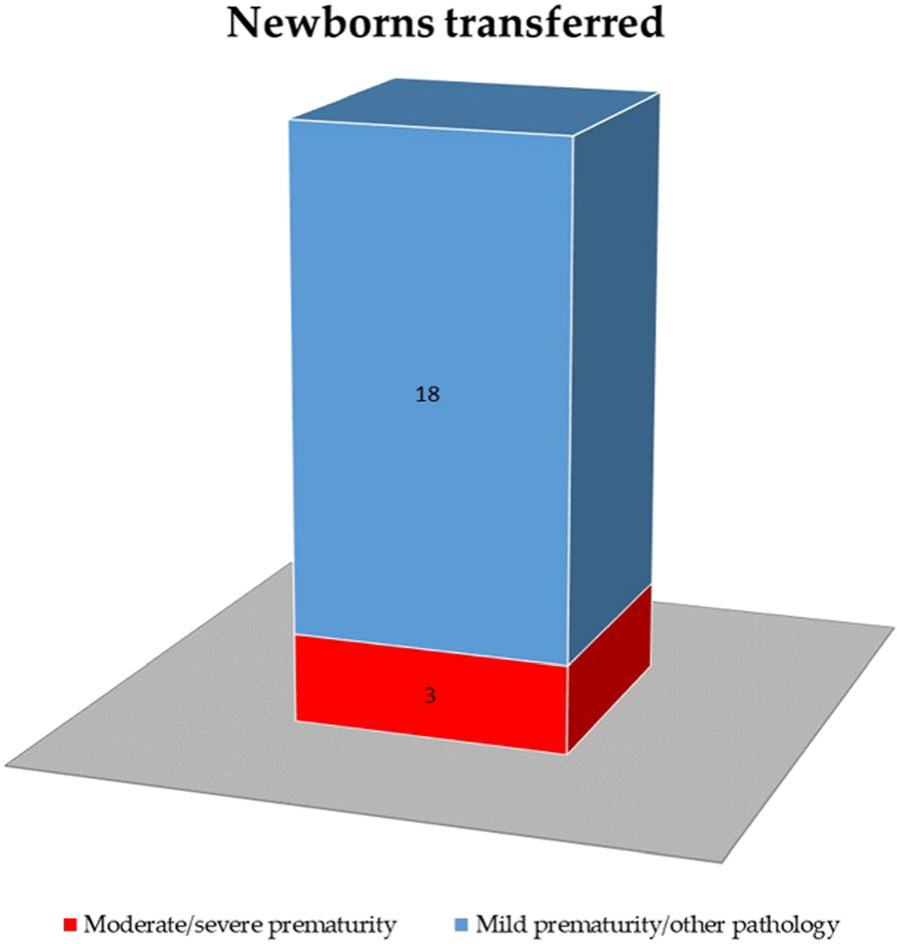
21 newborns (2.5%) were transferred to II level hospitals. Among them, 3 for moderate/severe prematurity, 18 for mild prematurity/other pathology

Twenty-one newborns (2.5%) were transferred to II level hospitals. Among them, 3 for moderate/severe prematurity, 18 for mild prematurity/other pathology (Fig. 4). A correlation between obstetric complications/non-physiological pregnancies and newborns transferred was found in 17/21 of them (81%), and no apparent association in 4/21 (19%). The obstetric pathological conditions observed were: placental abruption ± gestational hypertension/diabetes in 4 women (19%), fetal growth restriction ± tabagism 3 (14%), thrombophilia/thrombosis 3 (14%), preterm labor 2 (9.5%), puerperal infection/sepsis 2 (9.5%), fetal acute injury in otherwise physiological pregnancies 2 (9.5%).

**Fig. 5:**
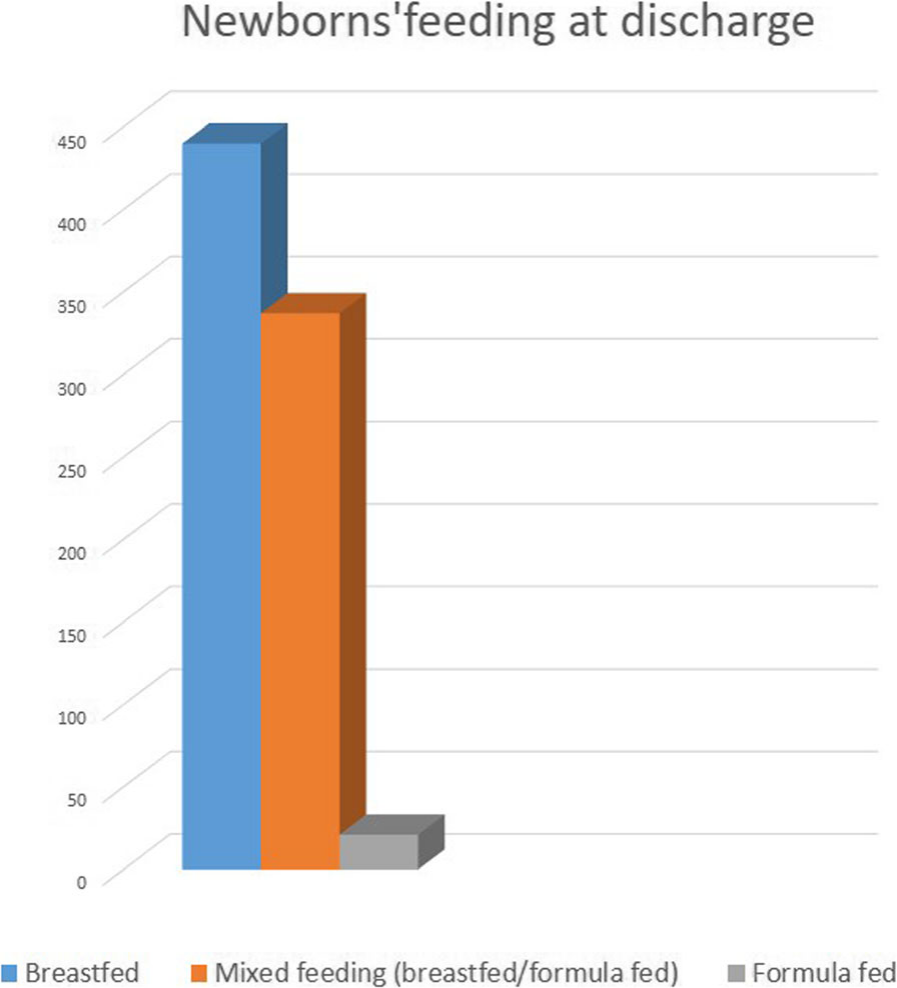
Of the 798 remaining newborns at discharge (out of the 822), 440 were breastfed (55%), 337 had a mixed feeding (breastfed/formula fed, 42%) and 21 were formula fed (3%)

No neonatal deaths occurred in the biennium under investigation, and the outcome is favorable to date for all the newborns transferred. Of the 798 remaining ones, 440 were breastfed at discharge (55%), 337 had a mixed feeding (breastfed/formula fed, 42%), and 21 were formula fed (3%) (Fig. 5), with overlapping incidence (53%) of breastfed among those born to mothers attending birth training courses.

## Discussion

The political and health reforms implemented in Italy during the last decades, and the increased economic well-being allowed the reduction of mortality rate of infants <5 years of age, from 346.5‰ in 1887 to 3.6‰ in 2015 [8]. Also mortality rates of infants <1 year and <1 week of age decreased, and in 2015 they were 3.1 and 1.4‰, respectively [9]. Despite an overall and continuous improvement in infant/neonatal mortality rates (IMR/NMR), with lower levels than those of several European countries, notable disparities still remain disadvantaging insular and southern regions (3.4‰) than center-northern ones (2.9 and 2.5‰, respectively) and foreign citizens (4.5‰) than Italians (2.6‰) [9].

The analysis of the main causes of mortality better defined the improvements obtained, revealing higher prevalence of congenital malformations and conditions of perinatal origin (69% in 2015), which progressively increased over time, than infectious diseases (2%), which conversely decreased [9]. Indeed, NMR drop from 1990 to 2014 by 40% worldwide, while perinatal mortality rate (PMR) only by 15%, reaching 4.1‰ in 2013 [10]. Variability among regions was observed also for PMR, showing higher values in Sicily (4.7‰), and lower in Lombardia (3.6‰) and Tuscany (2.7‰) [10].

The PMR found in our study, although it reflects a sample which is not entirely representative of the whole population of pregnant women (excluding certain risk categories), seems to be lower than national and regional data. Our results, indeed, underline that the PMR gap with northern regions and the national average may be filled. However, some critical issues emerge, especially those about the management of mild prematurity/pathology. Moreover, a correlation between obstetric complications/non-physiological pregnancies and newborns transferred to II level centers was found in most cases (81%). This highlights the continuity of care between mother and newborn, and the unpredictability of birth, whose complications are not prevented in all cases excluding maternal/fetal risk factors.

The quality of emergency obstetric and newborn care, rather than its simple availability, is then essential to prevent perinatal mortality/morbidity [11–14]. With the present analysis, we suggest that improvements in the observed critical areas may have a positive impact on mother and newborn health outcomes, and avoid many of maternal/fetal/neonatal deaths and diseases. Nevertheless, the measures to be taken often face challenges to scale up, many of which are context-specific [15].

Specifically, in accordance with literature data [16, 17], our results highlight the need to overcome obstacles as health workforce and financing [18]. Sufficient numbers of health care providers with specific competence, including trained midwives and neonatal nurses [19–21], may deliver quality care resulting in the best outcomes [12]. In fact, their employment is associated with more efficient use of resources, reduced mortality and higher quality of care for mothers and newborns. Such improvements should be, anyway, carried on within a multidisciplinary context, which includes also obstetricians, neonatologists and community health workers [12, 15].

High quality maternal and newborn health services should, then, require adequate financing. Conversely, the lack of investment in health is a well known issue [12, 22]. Various strategies have been employed in different countries to improve access to and utilization of maternity services, and they showed promising results [23–26]. Also in Italy, as underlined in the present report, such strategies should be applied, especially in southern regions and for complicated pregnancies and newborns with mild prematurity/pathology. Our findings specifically suggest that still HICs, mostly peripheral hospitals/rural areas, need to develop long term human resource plans for training and keeping health workers [27], particularly midwives, neonatal nurses [28], obstetricians and neonatologists [29–31]. Specific skills are needed for those caring for newborns with prematurity/pathology, and the lack of this specialized cadre in most settings is indeed consistent with our experience [32, 33].

Despite the critical issues detected, some encouraging results were obtained. We observed a low number (21, 2.5%) of newborns transferred on the total of live born, although among them those with mild prematurity/pathology were prevalent (18/21, 85%) (Fig. 4). Moreover, this data resulted in a milder impact than expected on “experience of care”, which is the patient’s perspective of the care received, for several presumable reasons.

First of all, the advantageous ratio between midwives and patients, which allowed human and care relationships of higher quality.

Secondly, a significant number of women (179, 22%) attended birth training courses with their partners. These took place during the study period, through regular meetings with all the figures of the staff, including clinical psychologists. They may have had not only a positive impact “*per se*”, but also an indirect reflection on the other mothers.

Finally, in the extra-urban and rural context like that here described, the hospital institution is deeply and intimately grafted onto the social network of the territory. The hospital and its operators, therefore, enjoy great credit and recognition at cultural and care level. The relationships between health workers and families, which have ancient roots, are unique and not dispersed and declined in the multiple health care offers, typical of the large urban centers and metropolitan areas.

The goal this study proposes, of improving the quality of care, could start by all the aspects above mentioned. The present setting, consolidating strengths and removing weaknesses, may represent a virtuous model, also in view of the strategic role played by the hospital in specific peripheral/rural contexts, such as the one here shown. The humanization and quality of care provided may, thereby, have favorable short and long-term effects on woman and child health. This is also supported by our rates of breastfeeding at discharge (55%, Fig. 5), higher than those of the whole Sicily in 2017–2018 (around 34%) [34]. The proposals to implement the services that peripheral centers can offer could, then, include: enhancement/enlargement of birth training courses with other professionals (i.e. dietician/nutritionist, cultural mediator, social worker) to the *postpartum* period, addressing obstetric and neonatal/pediatric issues (*postpartum* depression/maternity blues, puerperal hygiene, vaccinations, foreign body airway obstruction, complementary feeding); courses/dedicated outpatient services of breastfeeding support, facing the territory (i.e. home interventions) and integrated with the primary pediatric care; improvement of access to *peripartum* analgesia, respect for physiology, communication and sharing of health care procedures; free and active offer of prenatal screening tests to assess the risk of chromosomal abnormalities, aiming at reducing invasive diagnosis and centralizing high-risk pregnancies, also in support of the poorest population groups.

## Conclusion

Although the minimal standard of adequate perinatal care in Italy is >500 childbirths/year, the aims of the Italian legislation concern the rationalization of birth centers as well as the structural, technological and organizational improvement of health facilities [3]. However, reaching the minimal threshold of the reference legislation alone will not necessarily deliver the outcomes or achieve mortality/morbidity reduction targets. Therefore, specific contexts and critical areas need to be identified and managed [12]. Adequate resources and intervention strategies should be addressed not only to perinatal emergencies [35, 36], but also to the management of mild prematurity/pathology, especially in vulnerable populations for social or orographic reasons [37].

The reduction of the differences in health outcomes found among different population groups (i.e. for geographic area) are a dutiful and strategic commitment for most of health systems. The inequalities in infant/perinatal mortality rates are particularly serious [38]. The determinants of such disparities are complex to study and the interventions that can modify these causes and that may be translated into an effective decrease of inequalities can take a long time [39]. Nevertheless, the increasing availability and spread of health care offers, even in HICs, cannot be separated from the goal of quality of care, which is an ethic and public health imperative.

## Data Availability

The datasets used and analysed during the current study are available from the corresponding author on reasonable request.

## Abbreviations

FIVET: Fertilization in vitro with embryo transfer
GA: Gestational age
HICs: High-income countries
ICS: Intracytoplasmic sperm injection
IMR: Infant mortality rate
LICs: Low-income countries
NMR: Neonatal mortality rate
PMR: Perinatal mortality rate
UK: United Kingdom
USA: United States of America
WG: Weeks of gestation
